# Supplementary material for: End Sequence Analysis Toolkit (ESAT) expands the extractable information from single-cell RNA-seq data
Source: Genome Res. 2016 Oct;26(10):1397–410. doi: 10.1101/gr.207902.116 (PMC5052061; doi:10.1101/gr.207902.116)
Supplement: Supplemental Material [file supp_gr.207902.116_Supp_Materials.tar.gz › ESAT-esat_v0.1/doc/html/classumms_1_1core_1_1scripture_1_1_build_scripture_coordinate_space-members.html]

ESAT: Member List


ESAT


- Main Page
- Related Pages
- Packages
- Classes
- Files

- Class List
- Class Index
- Class Hierarchy
- Class Members

All Classes Namespaces Files Functions Variables Enumerator Pages

- umms
- core
- scripture
- BuildScriptureCoordinateSpace

umms.core.scripture.BuildScriptureCoordinateSpace Member List

This is the complete list of members for umms.core.scripture.BuildScriptureCoordinateSpace, including all inherited members.

|  |  |  |
| --- | --- | --- |
| BuildScriptureCoordinateSpace(File bamFile, String genomeDir) | umms.core.scripture.BuildScriptureCoordinateSpace |  |
| BuildScriptureCoordinateSpace(File bamFile, String genomeDir, String outputName, boolean forceStrandedness, TranscriptionRead st, ArgumentMap argMap) | umms.core.scripture.BuildScriptureCoordinateSpace |  |
| BuildScriptureCoordinateSpace(File bamFile, String genomeDir, String outputName, boolean forceStrandedness, TranscriptionRead strand, String chr, ArgumentMap argMap) | umms.core.scripture.BuildScriptureCoordinateSpace |  |
| compatible(Annotation assembly, Annotation read) | umms.core.scripture.BuildScriptureCoordinateSpace | static |
| getIsoformMap(Collection< Gene > genes) | umms.core.scripture.BuildScriptureCoordinateSpace | static |
| getOverlappingGenes(Gene gene, Collection< Gene > allGenes, double minPctOverlap) | umms.core.scripture.BuildScriptureCoordinateSpace | static |
| getOverlappingGenes(Gene gene, IntervalTree< Gene > allGenesTree, double minPctOverlap) | umms.core.scripture.BuildScriptureCoordinateSpace | static |
| getPaths() | umms.core.scripture.BuildScriptureCoordinateSpace |  |
| hasAnOverlappingGene(Gene gene, IntervalTree< Gene > allGenesTree, double minPctOverlap) | umms.core.scripture.BuildScriptureCoordinateSpace | static |
| isoformMapContains(Map< Gene, Set< Gene >> isoformMap, Gene gene) | umms.core.scripture.BuildScriptureCoordinateSpace | static |
| l2a(List< Double > list) | umms.core.scripture.BuildScriptureCoordinateSpace | static |
| main(String[] args) | umms.core.scripture.BuildScriptureCoordinateSpace | static |
| memoryStats() | umms.core.scripture.BuildScriptureCoordinateSpace | static |
| write(String save, Map< String, Collection< Gene >> rtrn) | umms.core.scripture.BuildScriptureCoordinateSpace | static |


---

Generated on Fri Aug 15 2014 15:11:14 for ESAT by  

 1.8.7
